# Reconsidering Hormone Replacement Therapy: Current Insights on Utilisation in Premenopausal and Menopausal Women: An Overview

**DOI:** 10.3390/jcm14207156

**Published:** 2025-10-10

**Authors:** Vesselina Yanachkova, Mariela Vasileva-Slaveva, Stoyan Kostov, Angel Yordanov

**Affiliations:** 1 Research Institute, Medica University Pleven, 5800 Pleven, Bulgaria; 2 Department of Endocrinology, Specialized Hospital for Active Treatment of Obstetrics and Gynaecology “Dr Shterev”, 1330 Sofia, Bulgaria; 3 Department of Breast Surgery, Specialized Hospital for Active Treatment of Obstetrics and Gynaecology “Dr Shterev”, 1330 Sofia, Bulgaria; 4 Department of Gynecology, Hospital “Saint Anna”, Medical University—“Prof. Dr. Paraskev Stoyanov”, 9002 Varna, Bulgaria; 5 Department of Gynecologic Oncology, Medical University-Pleven, 5800 Pleven, Bulgaria

**Keywords:** hormone replacement therapy, menopause, perimenopause, menopausal transition

## Abstract

Hormone replacement therapy (HRT) has been utilized in clinical practice for decades as a main therapeutic approach for mitigating menopausal symptoms. The symptoms mostly encompass vasomotor and genitourinary issues resulting from the deficiency of estrogen and progesterone. Initially identified as a universally advantageous and indispensable intervention, hormone replacement therapy subsequently became the subject of considerable scientific and clinical debate, especially after the publication of extensive epidemiological studies indicating potential adverse effects associated with cardiovascular and cancer risk. This study aims to reassess the role of HRT in clinical practice by analyzing its historical evolution, expanded clinical uses, and changes in guidelines necessitated by resent scientific studies. Current evidence from clinical studies and meta-analyses unequivocally demonstrates that hormone replacement therapy is the most efficacious treatment for vasomotor and urogenital symptoms, and also acknowledging its potential role in osteoporosis prevention. The administration of HRT requires careful individual assessment, considering the patient’s age, timing of initiation, existence of comorbidities. In this setting, therapy decisions have to be based on a combination of the most up-to-date clinical guidelines, risk stratification, and the patient’s preferences. In conclusion, the assessment of HRT confirms its primary role in reducing menopausal symptoms while also highlighting the imperative for a individual strategy that balances benefits and risks to improve outcomes for women.

## 1. Introduction

Hormone replacement therapy is a treatment for women undergoing or who have completed menopause, aimed at restoring hormone levels to their normative state. Throughout the menopausal transition and during menopause, there is a decline in estrogen and progesterone levels. Women experiencing perimenopause and menopause may find HRT advantageous, as it is a contemporary approach that mitigates the aging process and enhances quality of life. This therapy is widely recognized for its anti-aging benefits [1,2]. The primary objective of HRT is to alleviate menopausal symptoms associated with estrogen deficiency during the perimenopausal and menopausal phases. These symptoms include hot flashes, night sweats, vaginal dryness, mood swings, sleep disturbances, depressive episode, skin and metabolic changes. The long-term benefits of HRT include the prevention of metabolic abnormalities in lipid and carbohydrate metabolism, mitigation of increased cardiovascular risk, and preservation of bone density, all of which are consequences associated with reduced hormone levels. The initiation of HRT must be assessed by considering both the short-term and long-term benefits to the patient, as well as the associated short-term and long-term risks [1,2,3,4].

## 2. Historical Background

The discovery of hormone replacement therapy remains a highly debated topic. The elucidation of estrogen and progesterone’s roles in female physiology is credited to endocrinologists Allen and Doisy, who extracted estrogen from the urine of pregnant women in the 1920s. This groundbreaking work laid the foundation for understanding the regulation of the menstrual cycle in women of reproductive age and for recognizing the changes that occur during menopause. In 1943, Edgar Allen and Edward Doisy were awarded the Nobel Prize for their research and contributions to the study of sex hormones [5,6,7]. Twenty years later, the original oral estrogen therapy was introduced, derived from conjugated equine estrogens obtained from the urine of pregnant mares. In 1942, the FDA approved the pharmaceutical agent known as Premarin [8]. It was the first product of its kind and served as the most common estrogen therapy for a considerable time. Following the 1960s, hormone replacement therapy, primarily using estrogens, became essential for alleviating early menopausal symptoms. Observations in patients with an intact uterus, along with statistics on the occurrence of endometrial hyperplasia—a precursor to endometrial carcinoma—led to the development of combined estrogen–progestin therapy, marking the beginning of a new era in hormone replacement [9].

The implementation of hormone replacement therapy in clinical practice has generated various contentious viewpoints based on findings from clinical trials. The health risks associated with its use have raised concerns. The intensity of this issue has escalated following the release of data from the Women’s Health Initiative (WHI) study in the early 2000s. Results from this study indicated that certain groups of postmenopausal women who used combined hormone replacement therapy were at a higher risk of being diagnosed with breast cancer, heart disease, and strokes [10]. These findings have led to a significant decrease in the use of HRT and prompted a reevaluation of the risk-benefit ratio. However, despite the substantial patient sample, the study has several limitations. Key factors include the patients’ age at the initiation of HRT, the duration of treatment, and the dosage and method of administration. The primary cohort in the WHI consisted of women over 60 years of age, a demographic associated with increased risks for both cancer and cardiovascular events. Subsequent analyses emphasize the importance of initiating hormone replacement therapy within 10 years of the onset of menopause [11]. According to more recent literature, small amounts of HRT can be safely used until the end of life [1,2,3].

## 3. Menopause and the Menopause Transition—Both a Physiological Process and a Significant Risk Factor

**Figure 1:**
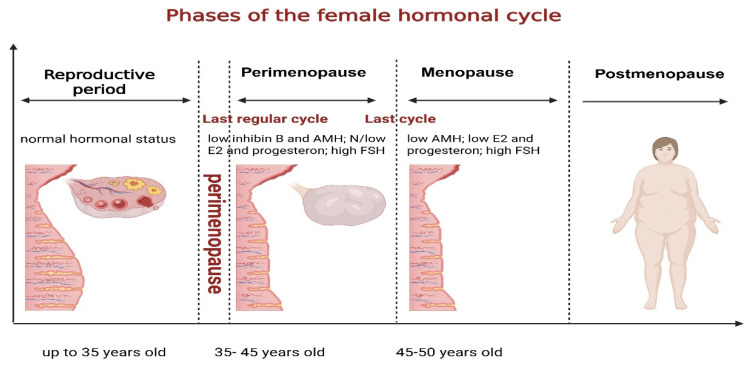
Phases of the female hormonal cycle. Created in https://BioRender.com.

The female hormonal cycle begins with puberty, advances through the reproductive phase, and ends with menopause (Figure 1). This process is continuously evolving and ongoing. The hormonal pathway is precisely regulated by the hypothalamic-pituitary-gonadal axis. Hormonal changes occur at each stage, from adolescence to menopause. Puberty marks the initiation of reproductive capabilities. During the reproductive phase, women experience regular menstrual cycles, which include distinct phases: follicular, ovulatory, and luteal. These phases are influenced by the activity of the pituitary-ovarian axis and the hormones follicle-stimulating hormone (FSH), luteinizing hormone (LH), estrogen, and progesterone.

The phase prior to menopause is known as perimenopause. It typically begins in a woman’s mid-forties and lasts for about 4 to 8 years. Indicators of this stage include hormonal instability, anovulatory cycles, irregular menstruation, and diminished ovarian reserve. During perimenopause, levels of inhibin B and anti-Müllerian hormone (AMH) decline, leading to increased levels of FSH and decreased levels of estradiol. These hormonal changes are associated with common symptoms such as hot flashes, night sweats, mood swings, and sleep disturbances [12]. Up to 80% of women experience vasomotor symptoms during this transition, which encompasses hot flashes and nocturnal sweating. A 2007 study by Freeman and colleagues indicates that mood disorders and cognitive deficits rank among the most prevalent psychological symptoms [13].

The transition to menopause is categorized into two phases in the STRAW (Stages of Reproductive Ageing Workshop) framework: the early phase and the late phase. The early phase is marked by changes in the menstrual cycle, while the late phase is characterized by the absence of menstruation and amenorrhea lasting at least sixty days [14].

For the purposes of this definition, menopause is identified as the cessation of menstruation for twelve consecutive months without any medical cause. Women typically experience this transition between the ages of 45 and 55. When menopause occurs, it signifies that the ovaries have ceased to produce eggs and can no longer support reproduction [15].

## 4. Types of Menopause

- Natural or physiological menopause constitutes a typical aspect of the ageing process. The typical onset age is approximately 51 years. This indicates that the ovaries cease functioning and producing estrogen over time, resulting in amenorrhea. To establish a diagnosis, it is essential to identify clinical neurovegetative symptoms and to have experienced amenorrhea for a minimum duration of one year. Regularly monitoring hormonal levels is inadvisable unless there is a suspicion of an underlying issue [1,2,3,15,16].
- Early menopause, occurring between the ages of 40 and 45, is diagnosed through clinical symptoms, particularly oligomenorrhea or amenorrhea persisting for over three months, and elevated FSH levels verified by two distinct measurements given within four to six weeks. FSH levels are not assessed throughout the use of birth control tablets by a woman [1,2,3,14,15,16].
- Premature Ovarian Insufficiency, or early menopause, occurs when menopause commences before the age of 40. It may be attributed to genetic factors, autoimmune disorders, or unidentified origins. Approximately 3.7% of women are affected by primary ovarian insufficiency [1,2,3,4,17,18]. As with early menopause, the diagnosis is clinical and laboratory: increased FSH levels.
- Induced Menopause—occurs due to medical procedures such as bilateral oophorectomy, chemotherapy, gonadotropin agonist or antagonist therapy, or pelvic radiation therapy. In contrast to natural menopause, the transition is fast, frequently resulting in more intense symptoms due to the quick decline in estrogen levels [1,2,3,15,18].

**Figure 2:**
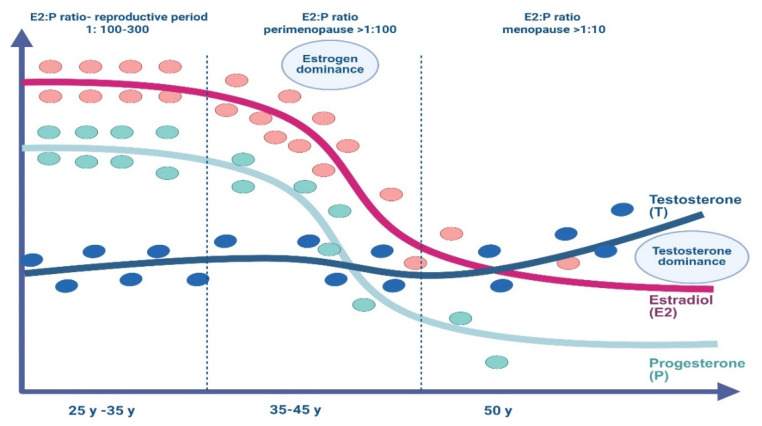
Alterations in the proportion of sex hormones during the menopausal transition and menopause. Created in https://BioRender.com.

Significant alterations in the concentrations and proportions of sex hormones transpire throughout menopause. These alterations substantially influence the emergence of clinical symptoms and underlie cardiometabolic consequences [1,2,3,4,13,16,17,19]. Following menopause, elevated bioavailable testosterone levels facilitate the shift from an estrogen-dominant to an androgen-dominant state. This implies that estrogen is no longer the primary hormone in the organism. This process continues for several years after menopause, during which the ovaries continue to produce an abundance of androgens. Despite the presence of low estrogen levels, the ovaries discharge an increased amount of androgens due to elevated gonadotropin levels. Levels of sex hormone binding protein diminish along with the reduction in estradiol levels (Figure 2) [20]. This worsens the estrogen-androgen imbalance by elevating the free androgen index. Visceral fat gain may occur due to increased bioavailable testosterone levels. The detrimental effects of androgens are more significant in visceral adipose tissue, correlating with a heightened risk of cardiometabolic illnesses, such as obesity, type 2 diabetes, cardiovascular disease, nonalcoholic steatohepatitis, and metabolic syndrome [21,22,23,24].

Changes in hormone levels in menopausal women are at the root of cardiometabolic complications. The decline in estradiol synthesis during menopause indicates that LDL cholesterol is not utilised for estradiol production, resulting in its accumulation in systemic circulation. Postmenopausal women exhibit elevated LDL cholesterol levels, which contribute to the development of metabolic syndrome, characterised by central obesity, insulin resistance, dyslipidemia, hypertension, and cardiovascular disease [21,22,23,24,25,26].

Surgical menopause is distinguished by a rapid decrease in estrogen and progesterone levels. In contrast to spontaneous menopause, which entails a gradual decline in ovarian function over several years, leading to the cessation of menstruation, the sudden drop in estrogen levels due to surgical removal of the ovaries is linked to rapid endothelial dysfunction, a significant increase in atherosclerotic lipoproteins and lipid oxidation, as well as an elevated cardiovascular risk, including the potential for myocardial infarction [21,22,23,24,25,26,27].

Hormone replacement therapy has a significant impact on neurovegetative symptoms and vaginal atrophy while also improving fat distribution, lipid metabolism, and insulin sensitivity in postmenopausal women. A meta-analysis by Salpeter et al. suggests that HRT positively affects the components of metabolic syndrome. HRT increases lean body mass and reduces abdominal fat, lowers HOMA-IR, and helps prevent the progression to diabetes in postmenopausal women without a prior history of diabetes. Additionally, HRT reduces fasting blood glucose levels and HOMA-IR in postmenopausal women with diabetes. The therapy also enhances metabolic functions by increasing levels of HDL-C and decreasing levels of LDL-C and fibrinogen in postmenopausal women, irrespective of diabetes status [1,2,3,19,20,21,22,23,24,25,26,27].

The primary cause of osteoporosis is menopause. During perimenopause and early postmenopausal, spinal bone mass diminishes as a result of elevated FSH levels and reduced estrogen production. Reduced bone mass and accelerated bone loss post-menopause elevate fracture risk in these women [1,2,3,25,26,27]. Postmenopausal osteoporosis is associated with diminished estrogen levels, which are essential for bone regeneration. Research indicates that 20% of bone loss transpires both prior to and during menopause. Trabecular bone density decreases by 50%, while cortical bone diminishes by 30% within the initial two decades following the loss of ovarian estrogen production. The spine is susceptible due to the high metabolic activity of the vertebrae’s trabecular bone, which considerably diminishes in the absence of estrogen. Fifty percent of postmenopausal women will have osteoporosis, and in many it will result in fractures. Osteoporotic fractures are linked to diminished mobility, decreased quality of life, and mortality [1,2,3,28,29,30,31].

Hormone replacement therapy preserves and enhances bone mineral density. Multiple studies indicate that hormone therapy commenced during perimenopause mitigates postmenopausal bone loss. Hormone therapy commenced at any age in postmenopausal women may confer advantageous advantages by mitigating additional bone loss [1,2,3,28,29,30,31].

## 5. When and What Hormone Replacement Therapy Should Be Prescribed?

Contemporary medical practice relies on a personalized approach to patient management. The menopausal transition and menopause are two distinct conditions that, despite presenting similar clinical symptoms, may necessitate different therapeutic strategies. The disparity primarily arises from variations in hormonal levels, specifically the quantitative requirements of hormones targeted in the therapeutic intervention. The initiation of hormonal changes is crucial, particularly during early menopause, as it influences the varying hormonal requirements and the degree to which a woman has fulfilled her reproductive objectives. Over time, studies have assessed the benefit-risk ratio and recommended HRT. The Menopause Society of North America (NAMS), National Institute for Health and Care Excellence (NICE), and the British Menopause Society (BMS) recommend safe stratification of hormone replacement treatment for women under 60 or within 10 years after menopause. HRT may provide minor risks to these women, but the benefits of reducing vasomotor symptoms and protecting bone health outweigh them. To treat menopausal symptoms, the guidelines recommend using the lowest effective dose for the shortest time. Women with long-term symptoms or osteoporosis risk should continue hormone replacement therapy. Clinical symptoms, side effects, and patient preferences should guide annual therapy re-evaluation.

Hormone replacement therapy is recommended for women experiencing premature ovarian insufficiency (POI) or early menopause resulting from surgical procedures or medical treatments. This enhances the management of menopausal symptoms [1,2,3,4,17,18]. In some cases, oral contraceptives are used for treatment for women experiencing premature menopause. Therapeutic intervention for these women should continue until natural menopause occurs.

It is important to acknowledge that HRT is not a contraceptive method. Women undergoing hormone replacement therapy during early menopause should be counseled to avoid conception for the following two years. The recommendations for women undergoing natural menopause regarding HRT indicate that pregnancy should be avoided for one year following menopause [1,2,3,4,18].

## 6. Hormone Replacement Therapy

Hormone replacement therapy is a treatment designed to restore declining levels of endogenous hormones (estrogen, progesterone, and, in certain instances, androgens). The NAMS Position Statement and the NICE guidelines recommend hormone replacement therapy for moderate to severe vasomotor symptoms, genitourinary syndrome of menopause, prevention or treatment of osteoporosis in high-risk women, premature ovarian insufficiency or early menopause (under 45 years), and induced menopause (post-oophorectomy) [1,2,3,4,32].

Hormone Replacement Therapy encompasses various combinations of estrogens, progestogens, and, occasionally, androgens or Selective Estrogen Receptor Modulators (SERMs). The specific needs of each patient are considered in the formulation of these combinations, which are subsequently tailored to meet those needs. Each hormone distinctly influences menopausal hormones [1,2,3,4] (Table 1).

The determinants of the various options for hormone replacement therapy encompass the specific hormone employed, the method of administration, the patient’s age, any existing comorbidities, and whether the woman has had a hysterectomy or possesses an intact uterus.

Estrogen is an important factor for regulation of vasomotor symptoms—hot flashes, and night sweats, vaginal atrophy as well as in the prevention of osteoporosis. Free estrogens, protein-bound estrogens, and conjugated estrogens are the three unique types that can be found in the human body. They are largely associated with proteins. Approximately 38–39% of them are tied to sex-hormone binding globulin (SHBG), 60% are attached to albumin, and only one to two percent are free estrogen. Estrone, 17β-estradiol, and estriol are the three primary natural estrogens that are produced by the body. Estriol is a metabolite of both estradiol and estrone, however it has limited hormonal activity. Estradiol and estrone are both capable of being converted into estriol. One of the most common forms of estrogen that is found in circulation is 17β-estradiol. [33].

The estrogen component in hormone replacement therapy can either be synthesised or derived from natural sources. The main types of estrogen include 17β-Estradiol, conjugated equine estrogens, and ethinylestradiol. 17β-Estradiol is a physiologically active form of estrogen that plays a crucial role in the female hormonal cycle and is the primary synthetic component used in HRT. It is a bioidentical formulation of natural estradiol. In clinical practice, it is available in various forms, including oral pills, transdermal patches, gels, and vaginal formulations. Additionally, it is included as an ingredient in several oral contraceptives. Following oral administration, it undergoes hepatic metabolism to estrone and various conjugates, such as sulfate and glucuronate, which are then excreted in feces and urine [33].

Estradiol exhibits a comparatively low bioavailability of approximately 5% upon oral ingestion, with individual variations. Approximately 95% is metabolised by first-pass metabolism in the liver to estrone and estrogenic conjugates. Consequently, the concentrations of circulating estrone and its conjugates markedly elevate the physiological ratio of estradiol to estrone, which is 1:1 for premenopausal women. This physiological ratio is particularly noticeable when transdermal formulations are used. In contrast, the ratio for oral administration of estradiol ranges from 1:5 to 1:20, resulting in a significant elevation of the metabolites [29,30,31,32,33,34]. With the oral administration of 1 mg of estradiol, levels of estradiol and its metabolites peak approximately 4 h after ingestion and maintain a constant level for a variable duration, ranging from 4 to 12 h. Following daily administration for a minimum of 2 weeks, a steady state is reached, resulting in the observation of a relatively constant concentration [33,34,35,36,37,38].

The initial metabolism in the liver and the alterations in metabolite ratios are associated with effects on blood coagulation factors and lipid metabolism. Parenteral administration of estradiol leads to a higher concentration as it bypasses hepatic metabolism, resulting in improved levels at equivalent doses compared to oral formulations. Transvaginal administration avoids first-pass metabolism in the liver, thereby promoting more stable hormone levels and diminishing the thromboembolic risk associated with their delivery. A vaginal injection of 0.5 mg of 17β-estradiol can produce concentrations up to ten times higher than those reached with 2 mg of estradiol taken orally [33,34].

Conjugated equine estrogens, commonly known as CEE, are derived from the urine of pregnant mares and include compounds such as equilin and estrone sulphate. When administered orally, these estrogens are rapidly absorbed into the bloodstream. At a dosage of 1.25 mg, they can reduce plasma FSH levels by 55% and LH levels by 40%. Conjugated equine estrogens exhibit a potency that is 1.4 to 3.5 times more effective than other forms of estrogen [33,34].

Ethinylestradiol is a synthetic estrogen that is effective when taken orally. This estrogen demonstrates stability during first-pass hepatic metabolism, remaining unaltered and retaining its action in circulation. In contrast to estradiol, ethinylestradiol has a high oral bioavailability of approximately 45% and exhibits a capacity for action that is 80 to 200 times greater, leading to a more pronounced hepatic influence throughout metabolism, with diminished fluctuations in hormone levels. Ethinylestradiol is infrequently used in hormone replacement therapy due to its high dosages, prolonged duration of action, and increased thrombotic risk. It is more commonly employed as a component in oral contraceptives, which necessitate higher estrogen dosages owing to the unique mechanism of action of these contraceptives [36,37].

Estrogen therapy may be linked to some adverse effects, which typically diminish within 3 to 6 months of initiation. The most prevalent symptoms include fluid retention, nausea, breast discomfort, bloating, leg cramps, headache, and occasionally vaginal bleeding. In instances of more pronounced or enduring adverse effects, dosage reduction or drug alteration is contemplated [1,2,3,4].

Progesterone is synthesized by the corpus luteum in the ovary and the adrenal gland. Progesterone circulates in the serum bound to cortisol-binding globulin, bound to albumin, or free. Upon entering circulation, it binds to progesterone receptors as well as to receptors for estrogen, androgens, and corticosteroids. This binding is crucial for its activities and clinical and therapeutic effects, including progestational, antiestrogenic, antiandrogenic, and anti-mineralocorticoid properties. Natural progesterone, at therapeutic doses, does not exhibit an antigonadotropic effect. Progesterone exhibits antimitotic and antiproliferative effects on the endometrium. Research indicates that treatment with estrogen alone results in glandular hyperplasia of the endometrium in 20–30% of cases [38,39,40].

The combined administration of estrogen and progesterone for a duration of 10 to 12 days can reduce endometrial hyperplasia from 2% to 0%. Progesterone activates 17-beta-estradiol dehydrogenase, leading to the conversion of estradiol to estrone, which is one of progesterone’s anti-estrogenic effects. It reduces the density of estradiol receptors in target tissues. The anti-androgenic effect is demonstrated through the stimulation of 5-alpha reductase, which inhibits testosterone metabolism. Progesterone also exhibits a natriuretic effect through its interaction with aldosterone receptors. A deficiency of progesterone during the latter half of the menstrual cycle is associated with fluid retention [39,40,41].

Progestogens are classified into two types: natural and synthetic. Progesterone is the only natural progestogen. In contrast, there are various progestins available for therapeutic use, which display considerable variation in their chemical structures. Progestins can be divided into two categories: (1) those that are structurally similar to progesterone and (2) those that are structurally similar to testosterone. Their effects vary in terms of antiandrogenic, antiestrogenic, and antiglucocorticoid properties. Derivatives of progesterone include dydrogesterone, hydroxyprogesterone, and medroxyprogesterone acetate, which vary in their half-lives and pharmacological activities. Derivatives of testosteorne encompass norethisterone, levonorgestrel, desogestrel, gestodene, gestronol, and norgestimate [39,40,41].

Synthetic progestins exhibit increased activity when administered orally. Compared to progesterone, they possess antiestrogenic, antiandrogenic, and antimineralocorticoid properties, while also suppressing the production of LH and prolactin, and occasionally normalizing the LH:FSH ratio. Synthetic progestins display a diminished anabolic effect; however, at elevated doses, they adversely influence lipid metabolism. Their antimineralocorticoid effect is markedly enhanced [39,40,41,42].

Micronised natural progesterone is bioidentical, lacks androgenic effects, and does not interact with androgen receptors. It demonstrates superior metabolic and cardiometabolic characteristics relative to synthetic formulations [1,2,3,4,39,40,41].

Drospirenone, a derivative of the synthetic aldosterone antagonist spironolactone, exhibits antimineralocorticoid activity, leading to a decrease in sodium retention and a reduction in blood pressure. Additionally, it demonstrates partial antiandrogenic action, approximately 30% of that of cyproterone acetate. It is available in conjunction with ethinylestradiol for contraceptive purposes [39].

Combined estrogen-progestogen therapy is recommended for women with an intact uterus to reduce the risk of endometrial hyperplasia and carcinoma. The estrogen/progestin combination may be utilised in several regimens—cyclic or sequential therapy—entailing daily estrogen treatment with the addition of progestin for 10–14 days per cycle, or continuously, to prevent withdrawal bleeding [1,2,3,4,32,36,42].

In women who have undergone subtotal hysterectomy, where the uterine body is removed while the cervix remains intact, the selection of hormone replacement therapy requires meticulous consideration. It is important to consider the presence of endometrial cells in the cervical canal, as treatment with estrogen alone may result in thickening of the endocervix. A test involving cyclic addition of progestin is warranted, as this therapy should correlate with the occurrence of withdrawal bleeding, thereby indicating the presence of endometrial cells [32,33,34,35,42,43]. Combination therapy effectively mitigates the risk of endometrial hyperplasia and subsequently decreases the likelihood of endometrial carcinogenesis.

Tibolone is a synthetic steroid medication that demonstrates estrogenic, progestogenic, and androgenic properties. The benefits are especially prominent in menopausal women experiencing diminished libido. Tibolone features a number of advantages in comparison to other types of hormone replacement therapy, including the fact that it is simple to administer and does not result in withdrawal bleeding in postmenopausal women [1,2,3,43].

For more than eight decades, testosterone has been utilised in the treatment of symptoms associated with perimenopause and menopause in females. Testosterone supplementation is contemplated for women with diminished libido and overall fatigue. The available formulations include patches, gels, and implants for testosterone. There is increasing evidence supporting the use of customised testosterone dosages for enhancing sexual function, preventing osteoporosis, and safeguarding breast health [1,2,3,4].

Selective Estrogen Receptor Modulators can mitigate menopausal symptoms and protect bone health while avoiding stimulation of breast tissue and the endometrium [1,2,3,4].

Hormone replacement treatment comes in several pharmacological forms for use in contemporary clinical practice, such as oral tablets; vaginal—tablets, pessary, cream, ring; transdermal-gel, spray, cream; subcutaneous implant; intrauterine devices. The selection of an appropriate therapeutic regimen, encompassing type, combination, and route of administration, is primarily influenced by whether the patient possesses a uterus (Table 2, Table 3, Table 4 and Table 5) [1,2,3,4,40].

Progestogen treatment reduces hyperplasia by mitigating the effects of estrogens on the uterine mucosa. The consensus is that bioidentical micronised progesterone is the safest option, regardless of whether a sequential or continuous regimen is employed. However, synthetic progestins are a suitable alternative. Administering the appropriate quantity in accordance with recommended standards is crucial. In the continuous regimen, the average dose of micronised progesterone is typically between 100 and 200 mg per day. Sequential therapy involves administering the same dose for 12 to 14 consecutive days. Progestins may not be prescribed alongside vaginal estrogens, when they are used to treat genitourinary syndrome in women with an intact uterus or in those patients treated with a SERM. Hormone replacement therapy recommendations additionally suggest using levonorgestrel-releasing intrauterine devices to reduce the effects of estradiol to prevent endometrial hyperplasia (Table 4) [1,2,3,4].

## 7. Comparison of Hormone Replacement Therapy and Oral Contraceptives

Hormone Replacement Therapy and Oral Contraceptives both utilise estrogen and progesterone; however, their indications, hormonal composition, target populations, and associated risks vary significantly. Understanding these distinctions is essential for clinical decision-making and patient guidance.

Oral contraceptives inhibit ovulation by suppressing the hypothalamic-pituitary-ovarian (HPO) axis. Ovarian function typically ceases, and Hormone Replacement Therapy reinstates endogenous hormone levels while not suppressing the hypothalamic-pituitary-ovarian axis [44].

Oral contraceptives elevate levels of synthetic estrogens, primarily ethinyl estradiol, and synthetic progestins to inhibit ovulation. Hormone replacement therapy employs reduced doses of natural or bioidentical estrogen, specifically 17-beta estradiol, in conjunction with a progestin, such as micronised or medroxyprogesterone acetate. This treatment emphasises symptom relief and protection of the endometrium in women with intact uteri [45,46]. Oral contraceptives are utilised by women of reproductive age, specifically those between 15 and 45 years, for the purposes of preventing conception or addressing hormonal disorders. The American Society for Reproductive Medicine Practice Committee indicates that these methods suppress ovulation and alter the endometrial lining and cervical mucus. The American Academy of Paediatrics Committee on Adolescence indicates that these methods are utilised for non-contraceptive purposes, including menstrual cycle regulation, acne reduction, and the treatment of endometriosis and PCOS [47,48].

Oral contraceptives may be utilised until the onset of natural menopause, at which point hormone replacement therapy can be maintained. This is contingent upon the patient’s medical and hormonal status [1,2,3,4].

## 8. Hormone Replacement Therapy and Associated Risks

Hormone Replacement Therapy is the most effective treatment, serving as the gold standard for alleviating menopause symptoms, protecting against osteoporosis, and diminishing cardio-metabolic risk. Notwithstanding the established advantages, the utilisation of HRT has certain hazards, primarily with breast cancer, thrombosis, stroke, and endometrial cancer. The risk can be effectively handled by considering the patient’s age at the commencement of HRT, the existence of concurrent disorders, family history of cancer and thrombotic events, along with the duration of therapy. This serves as the foundation for the recommendations of prominent professional organisations, including NAMS, NICE, and the BMS [1,2,3]. An individualised strategy is essential when commencing HRT (Table 6).

Certain complications may be associated with estrogen-based hormone replacement therapy. Oral estradiol, through enterohepatic metabolism, may elevate coagulation factors II, VII, IX, and X while reducing antithrombin III levels. This may increase the risk of thrombosis by 2 to 3 times, particularly during the initial year of treatment [48]. High-risk patients, including obese women, individuals with thrombophilia, those with a family history of thrombosis, and smokers, may receive estradiol via transdermal administration. This method optimises estradiol levels, thereby preventing hepatic metabolism. Oral estrogen therapy and prothrombotic factors may elevate pro-inflammatory variables. This may lead to macrovascular complications such as stroke and myocardial infarction. The outcome is contingent upon the duration of treatment and the initiation date [49].

Estrogens stimulate the growth of glandular tissue in the mammary glands, contributing to the development of breast cancer. The addition of progesterone in treatment further enhances mitotic activity. This risk is believed to increase when estrogen consumption exceeds five years or when combination therapy lasts between three and five years. The danger decreases after therapy is ceased, although it persists for several additional years [50,51,52,53]. Unopposed estrogen therapy in women with an intact uterus raises the risk of endometrial hyperplasia by two to four times, thereby increasing the likelihood of endometrial cancer [53,54,55,56]. Long-term estrogen therapy may be associated with a heightened risk of ovarian cancer due to the ongoing stimulation of ovarian epithelial cells. Reports indicate a reduction in this risk following the cessation of therapy. The use of transdermal estradiol may help alleviate this risk [57]. Additionally, oral estrogens can increase cholesterol saturation in the gallbladder, thereby raising the likelihood of gallstone formation by 1.5 to 2 times compared to individuals not receiving this treatment [58].

The type of progesterone, whether natural or synthetic, along with the dosage and administration schedule, significantly affects its risk profile in hormone replacement therapy. Natural micronised progesterone demonstrates superior safety compared to synthetic progestins. The extended use of combination estrogen and progestin HRT is associated with a markedly elevated risk of breast cancer. Progestins are known to increase mammographic breast density, an important factor for cellular proliferation. Micronised progesterone reduces its effectiveness [44,45,46,59].

Research, including the WHI and other extensive observational studies, indicates that synthetic progestins enhance the effects of estrogen on breast tissue more significantly than natural progesterone [10,50,51].

Certain synthetic progestins may counteract the positive effects of estrogen on blood vessels by increasing LDL cholesterol levels and causing vasoconstriction. The WHI trial demonstrated that the simultaneous administration of estrogen and progestin increased the risk of coronary heart disease, especially among older postmenopausal women beginning treatment [9]. Micronised progesterone demonstrates greater positive effects on cardiac health [50]. Progestins have the potential to induce a prothrombotic state. Combined hormone replacement therapy regimens, especially those that incorporate certain synthetic progestins, are linked to an increased risk of venous thromboembolism [49,60].

Testosterone is utilized in hormone replacement therapy, particularly for women experiencing surgical or premature menopause, as well as for those with sexual drive problems or significant overall muscular weakness. Testosterone therapy may offer therapeutic advantages; nevertheless, it also poses considerable hazards, particularly when dosages surpass physiological levels or when the treatment is extended. Typically, testosterone is delivered as a gel for hormone replacement therapy in women. The predominant unfavorable effects are associated with the androgenic characteristics of testosterone. The systemic administration of testosterone is linked to negative outcomes for cardiovascular and metabolic diseases; behavioral changes, and in some cases higher breast cancer risk [1,2,3,4].

Tibolone has benefits in treating menopausal symptoms and preventing osteoporosis; however, it may pose specific risks due to its estrogenic, progestogenic, and androgenic actions. Studies show that women over 60 are at a higher risk for experiencing a stroke. Tibolone may increase the chance of breast cancer recurrence; therefore, its use is contraindicated in those with a previous history of the disease. Evidence suggests a slightly increased risk of endometrial cancer; however, it is still generally lower than that associated with estrogen therapy alone. The risks of cardiovascular and thromboembolic events are lower than those linked to traditional hormone replacement treatment, although they cannot be completely dismissed.

Despite the advantages in alleviating menopausal symptoms, the accompanying hazards of hormonal impact necessitate a tailored and refined approach to its application. The existence of specific risk factors and comorbidities may serve as relative or absolute contraindications to HRT.

Absolute contraindications encompass cases where HRT presents an intolerable risk and must be categorically avoided, save in rare instances and under stringent oversight by a specialist. This pertains to women with hormone-sensitive malignancies; those with current or prior breast or endometrial cancer; individuals experiencing unexplained vaginal bleeding until the etiology is determined and neoplastic origin is ruled out; women with active or historical thromboembolic disorders, such as deep vein thrombosis or pulmonary embolism, particularly when estrogen monotherapy is indicated; women with a history of cardiovascular disease or uncontrolled hypertension, pending evaluation by a specialist, as well as those with a prior vascular event, including ischemic stroke or myocardial infarction; women with active hepatic disease; and patients exhibiting hypersensitivity to the treatment [1,2,3,4,48,49,50,51,52,53,54,55].

Relative contraindications provide reassessment following meticulous review of the benefit–risk ratio. An instance is a migraine, particularly when accompanied by an aura. It is frequently associated with premenstrual syndrome and is exacerbated by the effects of estrogens. In these instances, it is most efficacious to avoid systemic estrogen and instead employ transdermal administration at the lowest viable dosage. Arterial hypertension constitutes a relative contraindication, particularly when it is effectively managed. Certain women administering oral estradiol exhibit elevated systolic blood pressure. Hypertriglyceridemia and gallbladder diseases are regarded as relative contraindications. Hormone replacement therapy may exacerbate uterine conditions such as fibroids and endometriosis; therefore, a comprehensive evaluation is essential prior to initiation [1,2,3,34,57,58].

Hormone replacement therapy is still a key treatment for menopausal symptoms and a way to lower the risk of osteoporosis. Healthcare providers must meticulously tailor the commencement of HRT, the choice of administration method (oral, transdermal, vaginal, etc.), the dosage, and the duration of therapy, taking into account the patient’s clinical status, age, concurrent illnesses, and preferences. The primary focusses upon which HRT treatment should be based, according to established clinical practice recommendations and generally accepted guidelines, are as follows [1,2,3,17,18].

- The individualised approach—entails a meticulous evaluation of the benefit-risk ratio for each patient.
- The minimum effective dose—involves administering the least amount for the shortest duration possible. The selection of shape is based on the patient’s characteristics, comorbidities, and preferences.
- Evaluation of contraindications—includes a history of thrombosis or current thrombosis, a history of breast cancer, hormone-dependent neoplasia, uncontrolled hypertension, or hepatic dysfunction.
- Treatment monitoring—involves an annual assessment of the results and the need to continue the treatment.

Transdermal and vaginal hormone replacement therapy, by bypassing the hepatic metabolic process, present a more advantageous risk profile, particularly for patients with elevated vascular or thrombotic risk. Oral formulations are appropriate for women at low risk, possessing adequate liver function and lacking contraindications to treatment.

## 9. Methodology for Selecting the Appropriate Type of Hormone Replacement Therapy

1. In the presence of the uterus, the potential regimens for combined oestrogen and progesterone therapy are as follows:
  - Cyclic regimens are preferred for perimenopausal women.
  - Long-term combined therapy in postmenopausal women
2. In cases where the uterus is absent, estrogen-only therapy is typically initiated at a low dose and adjusted as necessary.

The selection of hormone replacement therapy should consider the age at which menopause begins, the presence of menopausal symptoms, and any underlying risk factors.

- Premature ovarian failure is managed with hormone replacement therapy, typically in a cyclic regimen until the onset of natural menopause.
- Perimenopause—commencement of cyclic combined therapy utilising estrogen and progesterone.
- Natural menopause occurs when HRT is initiated before the age of 60 and within 10 years of onset.
- Elevated risk of venous thromboembolism associated with transdermal combination therapy E + P
- Genitourinary syndrome involves the use of vaginal formulations of estradiol and dehydroepiandrosterone (DHEA).

## 10. Assessing the Impact of Hormone Replacement Therapy

- Three months post-initiation of hormone replacement therapy: assessment of symptoms, side effects, arterial pressure regulation, and observations regarding genital bleeding.
- Annual assessment of the risk-benefit ratio: risk evaluation, examination by a mammologist, and laboratory analysis of metabolic indicators.

## 11. Termination of Hormone Replacement Therapy

- The duration of treatment is not fixed; it is determined on an individual basis.
- In genitourinary syndrome, vaginal estradiol may be utilised for extended durations.
- A gradual reduction in the dosage is advisable.

## 12. Conclusions

Hormone replacement therapy is regarded as the gold standard for managing and controlling moderate to severe menopausal symptoms, including vasomotor and urogenital disorders, as well as for osteoporosis prevention. Reservations regarding the utilisation of hormone replacement therapy persist throughout scientific communities. In recent decades, studies have shown considerable advantages, especially when used in a balanced and tailored manner. To ensure that the benefits of this therapy outweigh its associated risks, it is necessary to consider the timing of initiation, have a comprehensive understanding of risk factors, and implement ongoing prophylaxis as fundamental aspects in achieving this goal.

## Figures and Tables

**Table 1:** Symptoms influenced by hormones in perimenopausal/menopausal women-positive effects: Created in https://BioRender.com.

Estrogen	Progesteron	Testosteron
Improves vasomotor symptoms Vulvovaginal atrophy Vaginal dryness Alleviate dyspareunia Sleep disturbances Improves lipid profile Reduce cardiovascular risk Maintain bone density Reduce osteoporosis and fracture risk Improves cognitive retention Influence depressed states Improves suppleness of skin and hair	Reduce risk of endometrial hyperplasia Reduce risk of endometrial carcinoma Reduce anxiety Sleep disturbances Improves cognitive retention Influence depressed states Reduce fluid retention Redue breast tension During menopause transition can regulate menstrual cycle	Improves sexual desire Improves orgasm Improves vaginal dryness Reduce fatigue Sleep disturbances Improves metabolism Reduce cardiovascular risk Maintain bone density Reduce osteoporosis risk Improves muscle strength Improves cognitive retention Influence depressed states

**Table 2:** Types of Hormone replacement therapy. Created in https://BioRender.com.

Type of Therapy	Hormones	Indications	Rout of Administration
Estrogen only HRT	Estrogen (E2)	Women without uterus	Oral, transdermal, subdermal, implants
Vaginal estrogen	Estrogen (E2	Urogenital symptoms	Vaginal tablets, cream, ring
Combined HRT: Sequential combined Continues combined	E2/progesterone P E2 daily/P intermittently E2/P daily	Women with uterus Perimenopause/early menopause Postmenopause	Oral, transdermal, vaginal
Tibolone HRT	E2/P/androgenic properties	Postmenopause	Oral
Testosterone NRT	Testosterone	Low libido, peri- postmenopause	Tnansdermal-cream, gel
Dehidroepiandrosteron DHEA	Supplement DHEA	Vaginal atrophy	Oral, vaginal
Bioidentical hormones	E2/P/testosterone	Not regulated by FDA	Oral, transdermal, vaginal

**Table 3:** Estrogen-based hormone replacement therapy formulations utilised in clinical practice.

Brand Name	Hormone	Dose	Route of Administration
Alora	Estradiol	0.05 mg daily patch	transdermal
Bissel	Estriol	50 mkg gel	vaginal
Climara	Estradiol hemihydrate	50 mkg patch	transdermal
Divigel	Estradiol	0.15 gel	transdermal
Estrofem	17-beta estradiol	1 mg, 2 mg	oral
Esrace	Estradiol hemihydrate	2 mg tablete	oral
Estring	Estradiol	2 mg ring	vaginal
Estrogel	Estradiol	0.06% gel	transdermal
Evorel	Estradiol	25/50/75/100 mkg patch	transdermal
Estradot	17-beta estradiol	100 mkg patch	transdermal
Evamist	Estradiol	1.53 mg dose spray	transdermal
Femseven	Estradiol	50/75/100 mkg patch	transdermal
Gynest	Estradiol	0.01% crem	transdermal
Lenzetto	Estradiol	1.3 mg dose spray	transdermal
Ovestin	Estriol	0.5 mkg pessarie; cream	vaginal
Proginova TS	Estradiol	50 mkg patch	transdermal
Progynova	Estradiol valerat	1 mg; 2 mg tablets	oral
Riselle	Estradiol	25 mg implant	subdermal implant
Sandrena	Estradiol	0.5 mg gel	transdermal
Vagifem	Esradiol	10 mkg tablets	vaginal
Vagirux	Estradiol hemihydrate	10 mkg vaginal tablete	vaginal
Vivelle-Dot	Estradiol	0.1 mg patch	transdermal
Zumenon	Estradiol hemihydrate	1 mg tablets	oral

**Table 4:** Progestin-containing hormone replacement therapy formulations.

Brand Name	Hormone	Dose	Route of Administration
Duphaston	Dydrogesterone	10 mg tablet	Oral
Gepretix	Micronized progesteron	100; 200 mg soft caps	Oral
Intrarossa	Progesteron	6.5 pessary	Oral
Mirena	Levonorgesterel	20 mkg per 24 h	Intrauterine device
Prometriom	Micronized progesterone	100 mg tab. 100; 200 mg vag.caps.	oral; vaginal
Primolut nor	Noretisterone	5 mg tablet	Oral
Provera	Medroxypogesteron acetate	5 mg tablet	oral
Utrogesten	Micronized progesterone	100 mg tab 100; 200; 300 mg vag.caps	oral; vaginal

**Table 5:** Main combined hormone replacement therapy products utilised in practice.

Brand Name	Hormone	Dose	Route of Administration
Activelle	E + Norehtisterion	1 mg/0.5 mg; 0.5/0.1 mg tab 0.05 mg/0.25 mg per 24 h patch 0.05 mg/0.14 mg per 24 h patch	oral; transdetmal
Angeliq	E + Drospirenon	1 mg/0.5 mg tablet	Oral
Climara Pro	E + Levonorgestrel	0.045/0.015 mg per day patch	Transdermal
Drovelis	Estertrol + Drospirenon	3 mg/14.2 mg tab	Oral
Evorel conti	E + Norethisterion	3.2 mg/11.2 mg	Transdermal
Femoston	E + Dydrogesteron	2 mg/10 mg tablet	oral
Femoston conti	E + Dydrogesteron	1 mg/5 mg tablet	oral
Femseven conti	E + Levonorgestrel	50 mkg/7 mkg patch	Transdermal
Kilofem	E + Norethisterion	2 mg/1 mg tablet	Oral
Trisequens	E + Norethisterion	2 mg/1 mg tablets	oral
Tibolone	Comined E + P + A	2.5 mg tablet	Oral
Testosteone	Testosterone	1% gel	Transdermal

**Table 6:** Main risks influenced by hormones in perimenopausal/menopausal women: Created in https://BioRender.com.

Estrogen	Progesteron	Testosteron
Thromboembolic events Cardiovascular enents-stroke & myocardial infarction Breast cancer Endometrial hyperplasia Endometrial cancer Ovarian carcinoma Gallbladder disease	Breast cancer Cardiovascular diseases Thromboembolic events Anxiety Mood disturbances Depressive episodes	Androgenic effects Lipid profile disturbances Cardiovascular events Liver toxity Behavioral changes All the side effects are dose and long-term use dependent

## Data Availability

The authors declare that all related data are available from the corresponding author upon reasonable request.

## References

[1] North American Menopause Society (2022). The 2022 hormone therapy position statement of the North American Menopause Society. Menopause.

[2] NICE (2023). Menopause: Diagnosis and Management. National Institute for Health and Care Excellence. https://www.nice.org.uk.

[3] Hamoda H., Panay N., Pedder H., Arya R., Savvas M. (2020). The British Menopause Society & Women’s Health Concern 2020 recommendations on hormone replacement therapy in menopausal women. Post Reprod. Health.

[4] GM-HRT-Guidance-for-Menopause-Management. https://gmmmg.nhs.uk/wp-content/uploads/2023/03/GM-HRT-Guidance-for-Menopause-Management-final-v1.0-approved-for-GMMMG-website.pdf.

[5] Simpson E., Santen R.J. (2015). Celebrating 75 years of estradiol. J. Mol. Endocrinol..

[6] Allen E. (1924). The hormone of the ovarian follicle; its localization and action in test animals, and additional points bearing upon the internal secretion of the ovary. Am. J. Anat..

[7] Doisy E.A., Veler C.D., Thayer S.A. (1930). Isolation of the follicular hormone. J. Biol. Chem..

[8] Utian W.H. (2008). The estrogen elixir: A history of hormone replacement therapy in America. J. Clin. Investig..

[9] Smith D.C., Prentice R., Thompson D.J., Herrmann W.L. (1975). Association of exogenous estrogen and endometrial carcinoma. N. Engl. J. Med..

[10] Rossouw J.E., Anderson G.L., Prentice R.L., LaCroix A.Z., Kooperberg C., Stefanick M.L., Jackson R.G., Beresford S.A., Howard B.V., Ockene J. (2002). Risks and benefits of estrogen plus progestin in healthy postmenopausal women: Principal results from the Women’s Health Initiative randomized controlled trial. JAMA.

[11] Manson J.E., Aragaki A.K., Rossouw J.E., Anderson G.L., Prentice R.L., LaCroix A.Z., Chlebowski R.T., Howard B.V., Thomson C.A., Margolis K.L. (2019). Menopausal hormone therapy and long-term all-cause and cause-specific mortality: The Women’s Health Initiative randomized trials. JAMA.

[12] Santoro N., Randolph J.F. (2011). Reproductive hormones and the menopause transition. Obstet. Gynecol. Clin..

[13] Freeman E.W., Sammel M.D., Liu L., Gracia C.R., Nelson D.B., Hollander L. (2007). Hormones and menopausal status as predictors of depression in women in transition to menopause. Arch. Gen. Psychiatry.

[14] Harlow S.D., Gass M., Hall J.E., Lobo R., Maki P., Rebar R.W., Sherman S., Sluss P.M., de Villiers T.J., STRAW+10 Collaborative Group (2012). Executive summary of the Stages of Reproductive Aging Workshop + 10: Addressing the unfinished agenda of staging reproductive aging. Fertil. Steril..

[15] World Health Organization (1996). Research on the Menopause in the 1990s.

[16] Gold E.B., Bromberger J., Crawford S., Samuels S., Greendale G.A., Harlow S.D., Skurnick J. (2001). Factors associated with age at natural menopause in a multiethnic sample of midlife women. Am. J. Epidemiol..

[17] Nelson L.M. (2009). Primary ovarian insufficiency. N. Engl. J. Med..

[18] ESHRE Guideline (2024). Management of Women with Premature Ovarian Insufficiency. https://www.eshre.eu/Guidelines-and-Legal/Guidelines/Management-ofpremature-ovarian-insufficiency.aspx.

[19] Silva G.B., Pascucci J.A., Karim H., Kaur G., Lerma R.O., Mann A.K., Gnanasekaran S., Silva G.D.B. (2024). Influence of the Onset of Menopause on the Risk of Developing Alzheimer’s Disease. Cureus.

[20] Jerilynn C.P. (1998). Perimenopause: The Complex Endocrinology of the Menopausal Transition. Endocr. Rev..

[21] Lobo R.A. (2007). Surgical menopause and cardiovascular risks. Menopause.

[22] Ko S.-H., Jung Y. (2021). Energy Metabolism Changes and Dysregulated Lipid Metabolism in Postmenopausal Women. Nutrients.

[23] Jeong H.G., Park H. (2022). Metabolic Disorders in Menopause. Metabolites.

[24] Mumusoglu S., Yildiz B.O. (2019). Metabolic Syndrome During Menopause. Curr. Vasc. Pharmacol..

[25] Nappi R.E., Chedraui P., Lambrinoudaki I., Simoncini T. (2022). Menopause: A cardiometabolic transition. Lancet Diabetes Endocrinol..

[26] Ko S.H., Kim H.S. (2020). Menopause-Associated Lipid Metabolic Disorders and Foods Beneficial for Postmenopausal Women. Nutrients.

[27] Salpeter S.R., Walsh J.M.E., Ormiston T.M., Greyber E., Buckley N.S., Salpeter E.E. (2006). Meta-analysis: Effect of hormonereplacement therapy on components of the metabolic syndrome in postmenopausal women. Diabetes Obes. Metab..

[28] LeBoff M.S., Greenspan S.L., Insogna K.L., Lewiecki E.M., Saag K.G., Singer A.J., Siris E.S. (2022). The clinician’s guide to prevention and treatment of osteoporosis. Osteoporos. Int..

[29] Askari M., Lotfi M.H., Owlia M.B., Fallahzadeh H., Mohammadi M. (2019). Survey of osteoporosis risk factors. J. Sabzevar Univ. Med. Sci..

[30] Charde S.H., Joshi A., Raut J. (2023). A Comprehensive Review on Postmenopausal Osteoporosis in Women. Cureus.

[31] Buckinx F., Aubertin-Leheudre M. (2022). Sarcopenia in Menopausal Women: Current Perspectives. Int. J. Womens Health.

[32] Grow D.R. (2002). Metabolism of endogenous and exogenous reproductive hormones. Obstet. Gynecol. Clin. N. Am..

[33] Wang-Cheng R., Neuner J.M., Barnabei V.M. (2007). Menopause.

[34] Kuhl H. (2005). Pharmacology of estrogens and progestogens: Influence of different routes of administration. Climacteric.

[35] Borgelt L.M. (2010). Women’s Health Across the Lifespan: A Pharmacotherapeutic Approach.

[36] Stanczyk F.Z., Archer D.F., Bhavnani B.R. (2013). Ethinyl estradiol and 17β-estradiol in combined oral contraceptives: Pharmacokinetics, pharmacodynamics and risk assessment. Contraception.

[37] McConaghy N. (2013). Sexual Behavior: Problems and Management.

[38] Oettel M., Schillinger E. (2012). Estrogens and Antiestrogens I: Physiology and Mechanisms of Action of Estrogens and Antiestrogens.

[39] García-Sáenz M., Ibarra-Salce R., Pozos-Varela F.J., Mena-Ureta T.S., Flores-Villagómez S., Santana-Mata M., De Los Santos-Aguilar R.G., Uribe-Cortés D., Ferreira-Hermosillo A. (2023). Understanding Progestins: From Basics to Clinical Applicability. J. Clin. Med..

[40] Nagy B., Szekeres-Barthó J., Kovács G.L., Sulyok E., Farkas B., Várnagy Á., Vértes V., Kovács K., Bódis J. (2021). Key to Life: Physiological Role and Clinical Implications of Progesterone. Int. J. Mol. Sci..

[41] Gompel A. (2018). Progesterone, progestins and the endometrium in perimenopause and in menopausal hormone therapy. Climacteric.

[42] Harper-Harrison G., Carlson K., Shanahan M.M. (2025). Hormone Replacement Therapy. StatPearls [Internet].

[43] Mwalwanda C.S., Black K.I. (2013). Immediate post-partum initiation of intrauterine contraception and implants: A review of the safety and guidelines for use. Aust. N. Z. J. Obstet. Gynaecol..

[44] Hsiao S.M., Liao S.C. (2024). Effect of tibolone vs hormone replacement therapy on climacteric symptoms and psychological distress. J. Chin. Med. Assoc..

[45] Prior J.C. (2017). Women’s reproductive system as balanced estradiol and progesterone actions—A revolutionary model. Gynecol. Endocrinol..

[46] L’Hermite M. (2017). Bioidentical menopausal hormone therapy: Registered hormones (non-oral estradiol and progesterone) are optimal. Climacteric.

[47] Practice Committee of the American Society for Reproductive Medicine (2017). Hormonal contraception: Recent advances and controversies. Fertil. Steril..

[48] AAP Committee on Adolescence (2014). Contraception for adolescents. Pediatrics.

[49] Chlebowski R.T., Anderson G.L., Aragaki A.K., Manson J.E., Stefanick M.L., Pan K., Barrington W., Kuller L.H., Simon M.S., Lane D. (2014). Hormone therapy and venous thromboembolism among postmenopausal women. Front. Horm. Res..

[50] Rossouw J.E., Prentice R.L., Manson J.E., Wu L., Barad D., Barnabei V.M., Ko M., LaCroix A.Z., Margolis K.L., Stefanick M.L. (2007). Postmenopausal Hormone Therapy and Risk of Cardiovascular Disease by Age and Years Since Menopause. JAMA.

[51] Chlebowski R.T., Anderson G.L., Aragaki A.K., Manson J.E., Stefanick M.L., Pan K., Barrington W., Kuller L.H., Simon M.S., Lane D. (2020). Association of Menopausal Hormone Therapy with Breast Cancer Incidence and Mortality During Long-term Follow-up of the Women’s Health Initiative Randomized Clinical Trials. JAMA.

[52] Beral V. (2019). Breast cancer and hormone-replacement therapy in the Million Women Study. Lancet.

[53] Fournier A., Berrino F., Riboli E., Avenel V., Clavel-Chapelon F. (2007). Breast cancer risk in relation to different types of hormone replacement therapy in the E3N-EPIC cohort. Int. J. Cancer.

[54] Fournier A., Berrino F., Clavel-Chapelon F. (2008). Unequal risks for breast cancer associated with different hormone replacement therapies. Breast Cancer Res. Treat..

[55] Lethaby A., Farquhar C., Sarkis A., Roberts H., Jepson R., Barlow D. (2004). Hormone Replacement Therapy in Postmenopausal Women: Endometrial Hyperplasia and Irregular Bleeding.

[56] Beral V., Bull D., Reeves G., Wmen Study Collaborators (2005). Endometrial cancer and hormone-replacement therapy in the Million Women Study. Lancet.

[57] Collaborative Group on Epidemiological Studies of Ovarian Cancer (2015). Menopausal hormone use and ovarian cancer risk: Individual participant meta-analysis of 52 epidemiological studies. Lancet.

[58] Liu B., Beral V., Balkwill A., Green J., Sweetland S., Reeves G. (2008). Women Study Collaborators. Gallbladder disease and use of transdermal versus oral hormone replacement therapy in postmenopausal women: Prospective cohort study. BMJ.

[59] Greendale G.A., Reboussin B.A., Slone S., Wasilauskas C., Pike M.C., Ursin G. (2003). Postmenopausal hormone therapy and change in mammographic density. Ann. Intern. Med..

[60] Canonico M., Plu-Bureau G., Lowe G.D., Scarabin P.Y. (2010). Hormone therapy and risk of venous thromboembolism in postmenopausal women. BMJ.

